# The Hidden Price of Plenty: Oxidative Stress and Calorie-Induced Cardiometabolic Dysfunction

**DOI:** 10.3390/life15071022

**Published:** 2025-06-27

**Authors:** Luka Komic, Marko Kumric, Jelena Komic, Marion Tomicic, Tina Ticinovic Kurir, Marko Grahovac, Marin Mornar, Doris Rusic, Josipa Bukic, Josko Bozic

**Affiliations:** 1 Department of Family Medicine, Split-Dalmatia County Health Center, 21000 Split, Croatia; luka.komic@dz-sdz.hr (L.K.); jelena.kelam@dz-sdz.hr (J.K.); marion.tomicic@mefst.hr (M.T.); 2 Department of Pathophysiology, University of Split School of Medicine, 21000 Split, Croatia; marko.kumric@mefst.hr (M.K.); tticinov@mefst.hr (T.T.K.); josko.bozic@mefst.hr (J.B.); 3 Laboratory for Cardiometabolic Research, University of Split School of Medicine, 21000 Split, Croatia; 4 Department of Family Medicine, University of Split School of Medicine, 21000 Split, Croatia; 5 Department of Endocrinology, Diabetes and Metabolic Diseases, University Hospital of Split, 21000 Split, Croatia; marko.grahovac@mefst.hr (M.G.); marin.mornar@mefst.hr (M.M.); 6 Department of Pharmacy, University of Split School of Medicine, 21000 Split, Croatia; doris.rusic@mefst.hr; 7 Department of Laboratory Medicine and Pharmacy, Faculty of Medicine, Josip Juraj Strossmayer University of Osijek, 31000 Osijek, Croatia

**Keywords:** adiposity, metabolic cardiovascular syndrome, nutrition, nutritional physiological phenomena, obesity, stress, nitro-oxidative

## Abstract

Overnutrition is a predominant issue in contemporary society, increasing rapidly despite considerable progress in our comprehension of nutrition, the health consequences of different food categories, and the dangers linked to excessive calorie consumption. The pathways connecting obesity to associated disorders are intricate, although research has consistently identified oxidative stress as a principal facilitator of the progression of many diseases. In this paper, the synthesis of various reactive species at the molecular level is studied, and the influence of diet on their production is assessed, with a thorough examination of the cellular mechanisms involved. Furthermore, the correlation between oxidative stress and the development of cardiometabolic diseases is explored, highlighting the most recent and relevant research in the field.

## 1. Introduction

Over time, dietary habits have evolved differently based on various factors such as culture, climate, wealth, food availability, and many more. However, it has long been recognized that consuming an excessive amount of calories poses a health hazard, and the importance of proper nutrition was even emphasized by Hippocrates himself: *“Let food be thy medicine, and medicine be thy food.”* Today, with significant advancements in science, technology, and medicine, we have a better understanding of the nutritional value, composition, and health benefits of various foods but also of the hazards associated with excessive calorie intake. Unfortunately, the rising number of obese individuals and those with cardiometabolic diseases suggests that such knowledge is hardly translated into daily human life [1]. Despite broad public health awareness, behavioral and structural determinants continue to promote obesogenic dietary patterns.

Such dietary patterns seem to increase oxidative stress at the molecular level, disrupting the body’s homeostasis, causing cell dysfunction, DNA mutation, apoptosis, insulin resistance, and metabolic dysfunction, raising risk for diabetes mellitus (DM), cardiovascular diseases, and ultimately leading to premature death [2]. While oxidative stress is not the sole causal factor in the pathogenesis of these disorders, it represents a converging point where multiple dysregulated pathways intersect, including nutritional excess, inflammation, neurohormonal imbalance, and mitochondrial dysfunction. Mounting evidence identifies oxidative stress as a critical mediator and amplifier of cellular and vascular injury in the context of metabolic disease. However, the complex and context-dependent nature of oxidative processes also helps explain why therapeutic interventions aimed at broadly reducing oxidative stress have largely failed in clinical settings. This suggests that targeting oxidative stress as a unifying mechanism may require more nuanced and temporally and spatially precise approaches.

In this comprehensive review, we propose that oxidative stress is a central, though not exclusive, mechanistic link between chronic overnutrition and cardiometabolic diseases. We evaluate the current understanding of how excessive caloric intake contributes to the generation of reactive oxygen and nitrogen species, examine the downstream effects on vascular and metabolic systems, and discuss how these molecular disruptions shape the progression of common cardiometabolic conditions.

## 2. Oxygen—A Medal with Two Sides

During the emergence of life on Earth, living beings flourished in an atmosphere that was deficient in O_2_, meaning they were primarily anaerobic [3]. As life progressed on the planet and the air became more oxygenated, living beings had to adjust to the rising levels of oxygen by evolving from being strictly anaerobic to becoming facultative anaerobes and ultimately aerobes. They also transitioned from autotrophs and fermenters to phototrophs and heterotrophs, utilizing the newly available oxygen for a variety of intricate metabolic processes and efficient energy production [3,4,5]. Today, we are well aware that oxygen is necessary for a range of biological processes including the hydroxylation of proline and lysine side chains in collagen, the elimination of xenobiotics using cytochrome P450, the breakdown of purine nucleotides into uric acid, the restoration of prosthetic groups to flavin through oxidative decarboxylation and other redox reactions, and the establishment of a chemiosmotic gradient in mitochondria via the electron transport chain [6]. While the utilization of oxygen undeniably resulted in a significant advancement in evolution, and most organisms, including humans, cannot survive without it, its increased levels and the detrimental byproducts produced during oxygen metabolism cause permanent harm to our bodies.

In 1954, Gerschman et al. made the initial suggestion that oxygen radical production was the cause of oxygen’s negative effects [3,7]. Today, we understand that this involves not solely oxygen radicals but also other nonradical compounds that are produced during oxygen metabolism, all of which are encompassed in the term reactive oxygen species (ROS). They include superoxide anion (O_2_^−^), hydroxyl radical (OH^−^), peroxyl radical (ROO), alkoxyl radical (RO), organic hydroperoxide (ROOH), hypochlorous acid (HClO), hydrogen peroxide (H_2_O_2_), and singlet oxygen (^1^O_2_) [8,9,10,11]. The most highly ROS identified is the hydroxyl radical, which almost inevitably harms cells, and since it is so reactive, there is no specific scavenger for it [3]. However, there are specific antioxidant defenses against superoxide anion and hydrogen peroxide. Superoxide dismutase eliminates superoxide anion by significantly accelerating its conversion to hydrogen peroxide, which is then eliminated by glutathione peroxidase [3]. Hydrogen peroxide can also be converted to water and singlet oxygen by catalase in peroxisomes and peroxisomal oxidase enzymes [3]. Although singlet oxygen is considered an ROS, it is the least reactive in the group [3]. The containment of transition metal ions in states that do not trigger ROS generation is also an important antioxidant mechanism. In general, antioxidants can be categorized into enzymatic, such as superoxide dismutase, glutathione peroxidase, glutathione S-transferase, and catalase, or non-enzymatic, including several endogenous and exogenous substances such as glutathione, melatonin, chelating proteins, uric acid, thioredoxin, vitamins A, C, and E, selenium, and zinc, which are utilized to terminate pro-oxidants. Alpha-tocopherol, one of the vitamin E isomers, is an example of an antioxidant from the latter group that captures ROO within the membrane [3,8,11,12,13].

In addition to ROS, there are other reactive molecules known as reactive nitrogen species (RNS). These encompass all chemical compounds that originate from nitric oxide (NO), including peroxynitrite (ONOO^−^), peroxynitrate (O_2_NOO^−^), nitrite (NO_2_^−^), nitroxyl anion (NO^−^), nitrosoperoxycarbonate (ONOOCO_2_^−^), nitrogen dioxide (NO_2_), and dinitrogen trioxide (N_2_O_3_) [8,9,10,11].

Moreover, there are various other molecules that promote oxidation, which can be classified as reactive sulfur species (RSS), reactive carbonyl species (RCS), or reactive selenium species (RSeS). All of these, along with the previously mentioned ROS and RNS, are constantly engaged in a battle with the antioxidant system. The pro-oxidative and anti-oxidative activities in the body are generally in equilibrium, with a slight inclination towards pro-oxidants. However, when this balance is significantly disrupted in favor of pro-oxidants, it results in a state of oxidative stress. Numerous factors contribute to oxidative stress, including different toxins, illnesses, tissue damage, and different environmental factors, one of which is nutrition, especially an excess of calories [3,8,9,10,11].

## 3. Nutritional Excess and Oxidative Stress—A Pathophysiological Perspective

Overconsumption of calories has been demonstrated to be detrimental even during the prenatal stage. Maternal obesity and overconsumption of calories amplify oxidative stress and impact the prenatal growth of organs, particularly the cardiovascular system, and elevate the subsequent susceptibility to the emergence of metabolic and reproductive ailments in adulthood [11,14].

Nowadays, the eating habits of the majority are recognized for their abundant consumption of saturated fats, refined sugars, and protein derived from animals, along with a deficient intake of fiber derived from plants, which define the eating regimen known as the Western diet [2]. This type of diet is frequently associated with an overabundance of calories consumed, which in turn leads to higher amounts of substances entering the process of mitochondrial respiration. Mitochondria are the primary origin of ROS [8,15]. Mitochondria are involved in a variety of biological processes, including the biosynthesis of amino acids, nucleic acids, lipids, heme, and purines, as well as the regulation of thermogenesis, cell division, and programmed cell death. However, the primary function of the mitochondria is the synthesis of ATP through the processes of oxidative phosphorylation [15,16,17,18]. Following the procedure of digestion and absorption of carbohydrates, lipids and proteins, including the fundamental constituents of these macronutrients, specifically glucose and other monosaccharides, fatty acids, and amino acids, become accessible to cells. Their subsequent breakdown generates acetyl-CoA, a compound that enters the citric acid cycle and provides reducing equivalents, with NADH and FADH_2_ being the most significant of these. Subsequently, these proceed to the mitochondrial respiratory chain in the inner mitochondrial membrane where they undergo oxidation, transferring their electrons from complex I or II to complex IV, and ultimately resulting in the formation of ATP and H_2_O molecules [15]. Premature electron leakage from complexes I and II is the most important course of ROS production by the mitochondrial respiratory chain [19,20]. When electrons escape individually, they generate superoxide anion; when they escape in pairs, they generate hydrogen peroxide [21]. However, complexes I and II are not the sole origins of ROS. In addition to sites I_F_ and I_Q_ in complex I and site II_F_ in complex II, there are eight such additional locations in mammalian mitochondria. Locations O_F_, P_F_, B_F_, and A_F_ are found in the 2-oxoacid dehydrogenase complexes, while site III_Qo_ is in complex III. Furthermore, sites G_Q_, E_F_, and D_Q_, along with the previously mentioned II_F_, are associated with the Q-dependent dehydrogenases in the QH_2_/Q isopotential pool [21]. The proportion of electron movement that is redirected to ROS creation is documented to be 1–2% [22,23]. Nonetheless, it can fluctuate from 0.01% to 2% as it depends on various factors, one of which is the degree of substrate provision [15,21]. Researchers have indicated that metabolite levels that are directly linked to the complete caloric consumption of food have a substantial impact on the overall degree of oxidative stress because a greater level of metabolites results in more substrate and electrons for the mitochondrial respiratory chain, consequently leading to more electron leakage [8,11,21,24].

In addition to ROS, mitochondria are also a location where RNS are generated. It has been demonstrated that complexes III and IV of the mitochondrial respiratory chain are the sites of NO production, and NO subsequently interacts with ROS to create RNS [25]. Furthermore, excessive nutrient intake can lead to the generation of ROS in various cellular compartments such as membranes, the endoplasmic reticulum (ER), lysosomes, and peroxisomes [26].

A vast group of enzyme complexes bound to membranes, known as the NADPH oxidase (NOX/DUOX) family, is responsible for generating reactive oxygen species (ROS) on the membrane. Alongside NOX2, which is the exemplar of the whole NOX family, there are six additional human counterparts of the catalytic component of the phagocyte NADPH oxidase: NOX1, NOX3, NOX4, NOX5, DUOX1, and DUOX2 [26,27]. Two lipid bilayer proteins, p22^phox^ and gp91^phox^, as well as four proteins located in the cytosol, p47^phox^, p67^phox^, p40^phox^, and the GTPase Rac2 constitute the enzymatic complex referred to as activated NOX2 [28]. A majority of the NOX family members necessitate cytosolic components for complete activation. In the case of NOX2, one of these components is already specified as p47^phox^, which was discovered to be significantly elevated following the consumption of glucose in the mononuclear cell homogenates [26,27,28,29]. Excessive production of ROS by polymorphonuclear leukocytes and mononuclear cells has been noted not only in relation to glucose consumption but also in relation to consuming a meal abundant in proteins and fats [30]. It was additionally demonstrated that a 48 h fast noticeably impacted the decrease of ROS generation by polymorphonuclear leukocytes and mononuclear cells, along with the concentration of the p47phox enzyme [31]. Hence, it can be inferred that this is yet another way by which a genuine high-calorie mixed meal triggers the generation of ROS.

The ER is another cell component with a high level of ROS production, which occurs during the protein folding process via oxygenase and oxidase located in the ER, some of which are part of the previously mentioned NOX group whose specific members are also found in the ER [26]. While the specific influence of overnutrition on the emergence of oxidative stress in the ER has not been documented, an excessive intake of calories directly influences the occurrence of ER stress, as well as indirectly through the generation of ROS by mitochondria [32]. Excessive nutrients may enhance the function of ER oxidase or disrupt the protein folding machinery in an oxidative manner, potentially leading to increased generation of ROS. Excess substrate may hinder the endoplasmic reticulum’s ability to achieve complete folding, potentially leading to incomplete disulfide bond formation and increased leakage of reactive oxygen species [33]. A hallmark of ER stress is the accumulation of unfolded and improperly folded proteins in the ER cavity [34]. By activating the mammalian target of rapamycin complex 1 (mTORC1), it has been shown that the intake of glucose and fatty acids leads to ER stress. The mTORC1 complex seems to induce ER stress and functions as an important sensor for bioenergetic status and excessive nutrition [32,35]. The exact way in which mTORC1 triggers ER stress is still unclear, but it may achieve this by promoting additional protein accumulation in the ER through stimulation of new protein production [32,36]. In addition, by modifying the redox state of the ER lumen, the previously observed generation of ROS by mitochondria due to excessive food intake is a significant trigger of ER stress [32,34]. Redox change may entail the transfer of ROS from mitochondria to the ER via membranes associated with mitochondria, elevating oxidative stress within the endoplasmic reticulum. Alterations in calcium transport between the ER and mitochondria may exacerbate oxidative imbalances by influencing redox enzymes within the ER [37,38]. The ER and mitochondria are physically and functionally interconnected through mitochondria-associated ER membranes, and both play a crucial role in cellular calcium signaling and homeostasis [34,39]. Once inside the ER, ROS disrupts the calcium channels, leading to a large release of calcium into the cytoplasm which can then be uptaken by mitochondria. Through mitochondria-associated ER membranes enriched with inositol 1,4,5-trisphosphate receptor (IP3R), the increased calcium concentration in ER can further enter the mitochondria, causing pH changes that further stimulate ROS production in the mitochondria, which can subsequently disrupt ER protein folding and induce ER stress [32,34,40]. It has been shown that ER stress and oxidative stress often reinforce each other in a positive feedback loop, disrupting cellular function, leading to pro-apoptotic signaling activation, and contributing to an increasing number of clinical disorders [34,41,42]. This reciprocal relationship may involve stress-activated signaling pathways, including PERK, IRE1, and ATF6, from the unfolded protein response. These pathways, while attempting to restore ER homeostasis, can also upregulate pro-oxidant genes or mitochondrial stress responses, thereby amplifying ROS production. The persistent activation of these pathways may lead to an increased likelihood of apoptosis through CHOP induction or caspase activation [43,44].

Lysosomes, which represent highly acidic cellular organelles involved in breaking down extracellular or intracellular macromolecules, are also affected by a high-calorie diet [26,45]. The specialized lysosomal enzymes are responsible for the degradation of macromolecules. An enzyme known as acid alpha-glucosidase is responsible for converting complex sugars into glucose. Lysosomal acid lipase is responsible for the breakdown of fats. It breaks down triglycerides and cholesteryl esters to produce free fatty acids and cholesterol [26,45,46]. To ensure the functioning of these acidic degradative enzymes, a pH of approximately 4.8 is necessary. Lysosomes seem to have a redox cascade that generates ROS because of maintaining this pH, which is similar to the already mentioned mitochondrial electron transport chain [26]. We can state that a high-calorie diet results in an increased number of compounds for the mentioned catabolic reactions, and in a comparable manner to within the mitochondrion, it results in an intensified production of ROS, which ultimately results in a condition of oxidative stress.

The oxidation of long-chain and branched-chain fatty acids, polyamines, and amino acids, and production of phospholipid and bile acids are some of the processes in which peroxisomes, single membrane-bound cell components, are engaged [26,47,48]. They additionally have a crucial function in the metabolism of ROS since they are the primary origin of hydrogen peroxide generated by the activity of numerous FAD-dependent oxidoreductases participating in different peroxisomal metabolic activities, the most noteworthy of which is the β-oxidation of fatty acids, which generate not just hydrogen peroxide but also superoxide anion [26,48,49]. Peroxisomes feature an antioxidative defense mechanism that works to limit the potential of oxidative stress, such as catalase, which is responsible for transforming hydrogen peroxide into water [47,48]. However, the question is whether the antioxidant defense system is effective enough under circumstances of high-calorie consumption and enhanced oxidation of fatty acids.

The impact of a high-calorie diet on the condition of oxidative stress has been examined in various research studies involving animals and humans. Braz et al. discovered that overfeeding young Wistar rats resulted in impaired mitochondrial respiration and elevated levels of oxidative stress indicators in the hypothalamus [50]. Lalrinzuali et al. demonstrated that consuming a fermented pork fat diet with an excessive number of calories, high-fat content, and high levels of fatty acid methyl esters induced oxidative stress in Wistar rats [51]. Ghaddar et al. observed increased cerebral oxidative stress in zebrafish that were overfed [52]. It is crucial to note that the majority of studies examining oxidative stress parameters in the context of excessive nutrition have utilized animal models, which differ from humans in terms of lifespan, metabolic rates, immune systems, and antioxidant profiles. These studies were performed under controlled conditions, whereas human studies are less ideal due to various environmental factors, concomitant comorbidities, and individual subject variability. Boden et al. identified 38 proteins, including mitochondrial enzymes like superoxide dismutase 2, that were associated with the production or elimination of ROS and were upregulated in adipose tissue during overnutrition in men who consumed approximately 6000 kcal/day of a mixed diet (composed of around 50% carbohydrates, 35% fat, and 15% protein) [53]. However, Toledo et al. found that skeletal muscle mitochondria were highly resistant to nutrient overload and that overfeeding had no impact on the release of ROS [54]. It is conceivable that the aforementioned observations rely on the cellular subtype and undeniably necessitate further investigation. Nevertheless, it is noteworthy that in the Toledo et al. study, participants were provided with a caloric intake that was only 40% above the recommended level, whereas in the Boden et al. study, participants experienced a caloric intake that was 200–300% higher than the recommended level. Consequently, a 40% increase in caloric intake among healthy young individuals was likely to have been inadequate to surpass the antioxidative capacities that effectively managed excess reactive oxygen species intracellularly over the observed period. Furthermore, Ávila-Escalante et al., in their review of randomized controlled intervention trials focusing on dietary interventions in patients with metabolic diseases who exhibited in vivo markers of oxidative stress, concluded that there was substantial evidence indicating that dietary changes could influence redox status in overweight or obese individuals, as well as those with hypertension, DM, or dyslipidemia [8]. Several studies have indicated that a calorie-restricted diet is linked to reduced indicators of oxidative stress [8,55,56,57].

It is also important to note that genetic variants in antioxidant system genes may influence the effectiveness of the antioxidant system, potentially resulting in heightened oxidative stress and an increased risk of obesity and cardiometabolic comorbidities. Antioxidant enzymes, including glutathione peroxidase, catalase, superoxide dismutase, and paraoxonase, as well as ROS producers and transcription factors, possess genetic variants that influence their susceptibility to oxidative stress and its associated adverse effects [58]. Krishnamurthy et al. reviewed variations in prooxidant and antioxidant genes, highlighting the significance of identifying these genes in the context of personalized medicine [59]. Oxidative processes are also linked to epigenetics. The connection discussed is significant in relation to cardiometabolic diseases; however, the existing literature predominantly focuses on the relationship between oxidative stress and epigenetic changes within oncological contexts. Consequently, oxidative stress has been demonstrated to influence the regulation of noncoding RNAs, histone modifications, and DNA methylation [60,61]. Medicine is progressing towards a more individualized approach, incorporating genetic analysis to enhance the identification of vulnerable individuals and to expedite the diagnosis, treatment, and prevention of cardiometabolic diseases.

In summary, it can be concluded that excessive feeding increases the production of ROS and leads to oxidative stress through multiple mechanisms and in different cellular compartments. The detrimental effect of ROS is caused by various mechanisms, including oxidation and consequent DNA damage, activation of transcription factors, an increase in inflammatory factors, dysfunction of mitochondria, and damage to proteins and lipids, as well as alterations in epigenetics. Oxidative stress is linked to the emergence and advancement of numerous illnesses, with a significant portion of these being cardiometabolic disorders [2,26].

## 4. Overfeeding, Oxidative Stress, and Cardiometabolic Diseases—Connecting the Dots

Low amounts of ROS, which under physiological circumstances are equivalent to their elimination, are extremely important for the proper functioning of physiological cellular processes, cellular signaling and the immune system [62,63]. However, when ROS are present in abundance, such as in cases of overconsumption of calories, this results in a condition of oxidative stress that has various harmful impacts on virtually all organ systems. In the context of cardiovascular diseases, it is crucial to highlight the adverse effect of oxidative stress on the endothelial function, which leads to its impairment [64]. According to classical teaching, under regular circumstances, NO synthesized by endothelial nitric oxide synthase (eNOS) promotes vasodilation of blood vessels but more importantly exerts many vasoprotective and antiatherosclerotic effects [65,66,67]. In a condition of oxidative stress, NO is inactivated by interaction with superoxide anion; in addition to the inactivation of NO itself, creation of RNS occurs, particularly peroxynitrite, which in subsequent molecular interactions can induce a number of detrimental effects [65,67]. However, the narrative does not end there, because oxidative stress also has an impact on eNOS, leading to its uncoupling when it generates superoxide anion rather than NO [65]. Therefore, uncoupled eNOS is regarded as one of the key mechanisms of endothelial dysfunction because it greatly adds to vascular oxidative stress and creates a vicious cycle that causes a decline in NO production and an increase in ROS generation [65,68]. Aside from that, NO has multiple other vaso- and cardioprotective effects, which is why its downregulation contributes multiple cardiometabolic diseases that we discuss.

Oxidized LDL is another substance that plays a role in the decoupling of eNOS and the development of atherosclerosis, although its role in atherosclerosis pathogenesis has been challenged lately since scarce evidence supports the causal role of oxidized LDL in humans [69]. It is presumed that under the effect of oxidative stress, LDL is oxidized and then accumulated in macrophages, where it causes the formation of foam cells. It is believed that these foam cells emit several cytokines, which inflame and stress the vascular endothelium, thereby creating a vicious cycle that results in development of cardiovascular disease and eventually adverse cardiovascular events [64,70,71]. By oxidizing the key signaling phosphatases and kinases, as well as activating NF-κB, oxidative stress can also promote vascular remodeling and inflammation. This ultimately results in the proliferation and migration of vascular smooth muscle cells, which are among the main features of the atherosclerotic process [72,73]. Additionally, by oxidation of matrix metalloproteinases, ROS also encourages vascular remodeling. These zinc-dependent endopeptidases are crucial for blood vessel growth, remodeling, and angiogenesis and are unfortunately linked to various phases of atherosclerosis.

It has been found that the generation of NO is diminished by matrix metalloproteinase 2 (MMP-2) proteolytic degradation of eNOS and its cofactor heat shock protein 90 (HSP90). Additionally, MMP-2 interacts with the protease-activated receptor 1 on endothelial cells, which in turn increases the expression of the vascular cell adhesion molecule 1 (VCAM-1) and increases monocyte subintimal layer infiltration, which ultimately results in atherosclerosis [72,74,75,76,77]. From the foregoing, although atherosclerosis pathogenesis is an ever-changing landscape, it can be inferred that oxidative stress is connected via a variety of mechanisms to the emergence of endothelial dysfunction and atherosclerosis, which, depending on the location, results in a variety of repercussions such as coronary, cerebral, carotid, renal, and peripheral artery diseases. All of these have the potential to eventually result in adverse cardiovascular events, significantly reducing the quality of life of these patients and causing premature death.

Hypertension is typically representative of disease in which the effects of oxidative stress have been implicated as fundamental mechanisms in pathogenesis. As in most diseases, oxidative stress is a mediator, rather than the cause, since in the setting of hypertension, an increase in oxidative stress in fact reflects inappropriate activation and/or over-expression of endothelin-1, urotensin, catecholamines, RAAS, and multiple other endocrine and paracrine pathways. Urotensin II, a potent vasoconstrictor and pro-inflammatory peptide, is upregulated in response to oxidative stress, which in turn exacerbates oxidative stress and stimulates the production of pro-inflammatory cytokines via the activation of NF-κB, p38 MAPK, and IRF3 [1]. This results in enhanced vasoconstriction, endothelial impairment, and the activation of vascular remodeling. Clinically, increased levels of urotensin II are associated with indicators of oxidative damage and inflammation and have been noted in obese children and adolescents with metabolic syndrome, as well as in cases of obstructive sleep apnea, which is strongly linked to cardiometabolic disorders [78,79,80]. The disruptive activity of eNOS and reduced production of NO changes the balance of vasoconstriction and the vasorelaxant effect of NO, leading to the development of hypertension [68,72,81]. Moreover, NO itself opposes the effects of the aforementioned molecules (endothelin, angiotensin…), thus taking part in prevention of vascular remodeling which is a major factor in progression and unceasing nature of hypertension [82]. Additionally, as further discussed, multiple detrimental effects of ROS on renal function are implicated in the maintenance of hypertension, whereas its effects on cardiomyocytes describe the damaging effect of hypertension on cardiac function beyond BP values and consequent development of heart failure. Obesity is perhaps the most established risk factor for essential hypertension [83]. The presumed mechanisms underlying the association between obesity and hypertension are complex and entail interactions between renal, metabolic, and neuroendocrine pathways. Multiple human and animal models have demonstrated that sympathetic nerve activity (SNA) is upregulated in obese individuals; moreover, recent research indicates a correlation among gut microbiota, SNA, and obesity-related hypertension [83,84,85]. Moreover, relatively short-term overfeeding itself has been associated with upregulated SNA [86]. Although volume expansion and sodium retention would normally suppress RAAS in obese individuals, multiple reports indicate that multiple elements of RAAS (renin, angiotensin, aldosterone) are in fact upregulated in these patients [87,88]. The mechanisms behind such observations include SNS and RAAS interconnection, mineralocorticoid-secreting factors produced in adipocytes, and physical compression of the kidneys by visceral and retroperitoneal fat, which impedes kidney flow [89,90,91]. Finally, as supported by abundant data, hyperleptinemia and the development of insulin resistance further contribute to pathogenesis of hypertension in obese patients [92,93]. Overall, it can be concluded that development and progression of hypertension can be induced by both overfeeding and obesity, with oxidative stress playing an important connecting mechanism.

Oxidative stress also emerged as a major player in the development of insulin resistance and DM [94]. Normal insulin signal transduction is a complex process that begins with the binding of insulin to the α-chain of the insulin receptor on the cell membrane, after which a cascade of reactions is initiated that includes many mediators and enzymes such as insulin receptor substrates, SHC-transforming protein, APS protein, extracellular signal-regulated kinase 1/2, atypical protein kinase C, protein kinase B (Akt), serine/threonine-protein kinase 2, ribosomal protein S6 kinase beta-1, mammalian target of rapamycin (mTOR), rho-associated protein kinase 1, AMP-activated protein kinase, glycogen synthase kinase 3, phosphoinositide 3-kinase, phosphatidylinositol 4,5-bisphosphate (PIP_2_), phosphatidylinositol (3,4,5)-trisphosphate (PIP_3_), and glucose transporter type 4 (GLUT4) [94,95,96]. If any of these mediators are compromised, normal insulin signal transduction may be hampered, leading to insulin resistance and DM. As a result of oxidative stress, several molecular pathways such as insulin receptor substrate phosphorylation, activation of several serine-threonine kinase pathways like IKK-β, NF-κB, and JNK that lead to the degradation of insulin receptor substrates, decreased Akt phosphorylation, suppression of GLUT4 expression and localization in cell membrane, activation of inflammatory pathways, decreased insulin-induced insulin receptor substrate-1, and PIP kinase relocation between the cytoplasm and microsomes are all affected. Oxidative stress also alters the activity of AMP-activated protein kinase, mTOR, and glycogen synthase kinase 3, which together with the induction of the p38 MAPK pathway impede insulin signal transmission [94,97,98,99,100,101,102,103,104,105,106]. Another way that oxidative stress contributes to insulin resistance is by impairing beta cells. These beta-cell impairment mechanisms include upregulation of apoptotic processes and downregulation of metabolic pathways in beta cells, K_ATP_ channels impairment, inhibition of transcription factors such as Pdx-1 and MafA, diminished beta cell neogenesis, mitochondrial dysfunction, activation of toll-like receptors, and activation of some of the already mentioned molecular pathways (NF-κB, p38 MAPK, JNK/SAPK) [94]. Additionally, oxidative stress directly impacts GLUT-4. After using mitochondria-targeted paraquat (MitoPQ) to produce superoxide within mitochondria, Fazakerley et al. demonstrated that both adipocytes and myotubes showed reduced insulin-stimulated glucose uptake and impaired GLUT4 translocation to the plasma membrane [94,107]. Catestatin, a bioactive peptide derived from chromogranin A, has emerged as a vital endogenous regulator that antagonizes multiple oxidative and inflammatory pathways. It directly facilitates hepatic glycogen synthesis, augments fatty acid oxidation, diminishes gluconeogenesis and glycogenolysis, increases downstream insulin signaling, and prevents atherosclerosis and inflammation [108,109,110]. Research indicates that obese children and adolescents with metabolic syndrome exhibit reduced levels of catestatin, underscoring its crucial function in sustaining metabolic balance [111].

The relationship between overfeeding and oxidative stress remains complex and, at times, contradictory. While multiple studies report that overfeeding—particularly diets rich in saturated fats and refined carbohydrates—leads to increased ROS generation and impaired redox homeostasis, other studies have failed to detect a significant rise in oxidative stress markers following short-term or moderate caloric excess. These discrepancies can be partly attributed to differences in experimental design, such as the duration and intensity of overfeeding, the macronutrient composition of the diet, and the specific oxidative stress biomarkers measured. Furthermore, individual variability including differences in antioxidant capacity, metabolic flexibility, and baseline health status may influence the observed outcomes. It is therefore plausible that oxidative stress induced by overfeeding operates on a threshold model, where only prolonged or excessive nutrient surplus surpasses endogenous antioxidant buffering, thereby leading to measurable redox imbalance. Reconciling these findings highlights the need for standardized, longitudinal studies that integrate both metabolic and redox parameters to more precisely define the dose–response relationship between overnutrition and oxidative stress.

In 2013, Paulus and Tschöpe proposed a new paradigm for heart failure with preserved ejection fraction (HFpEF) development, by which a systemic proinflammatory state is induced by comorbidities (e.g., obesity, diabetes, hypertension), subsequently causing myocardial structural and functional alterations [112]. Specifically, the developed state causes coronary microvascular endothelial inflammation. A key consequence of this endothelial inflammation is the overproduction of ROS, which directly scavenge NO, leading to a marked reduction in NO bioavailability and content. NO is crucial for the preservation of vascular homeostasis and vasodilation; its depletion leads to increased coronary microvascular resistance and impaired endothelial-dependent vasodilation [113,114,115]. Moreover, diminished NO levels hinder the activation of soluble guanylate cyclase (sGC) in neighboring cardiomyocytes, resulting in decreased production of cyclic guanosine monophosphate (cGMP) and consequent reduction in protein kinase G (PKG) activity [116]. Reduced PKG activity directly impacts the large sarcomeric protein titin, which is essential for cardiac flexibility. Reduced PKG-mediated phosphorylation specifically results in titin hypophosphorylation, which elevates cardiomyocyte passive stiffness and hinders diastolic relaxation [117,118,119]. This rigidity leads to increased left ventricular filling pressure and the characteristic diastolic dysfunction seen in HFpEF. The intrinsic myocardial stiffness is exacerbated by external variables, including epicardial adipose tissue (EAT) and pericardial restriction, which further hinder ventricular compliance. The proliferation of EAT in HFpEF, particularly in obese individuals, serves both as a mechanical obstruction and as an active endocrine organ. Through its endocrine and autocrine secretion of pro-inflammatory cytokines, EAT promotes myocardial fibrosis, exacerbates coronary microvascular dysfunction, and imposes pericardial restraint, thus intensifying diastolic dysfunction and increasing ventricular stiffness [120,121,122]. Excess epicardial adipose tissue (EAT) contributes to an inflammatory state through the activation of M1 macrophages and suppression of M2 macrophages, which enhances pro-inflammatory cytokine production and nuclear NF-κB activation, promoting cardiac remodeling [123,124]. Free fatty acids from EAT penetrate cardiomyocytes, leading to lipotoxicity, mitochondrial dysfunction, and increased apoptosis, particularly through palmitic acid-induced stress mechanisms. These free fatty acids also promote the release of inflammatory adipokines while inhibiting adiponectin, thereby exacerbating myocardial damage [125,126]. Disruptions in the renin-angiotensin system, specifically ACE2 deficiency, increase EAT inflammation and lipotoxicity, which contribute to impaired diastolic function and a heightened risk of HFpEF [123]. Furthermore, pulmonary vascular remodeling, triggered by sustained elevations in left atrial pressure, increases pulmonary artery stiffness and resistance, which further burdens the right ventricle, ultimately leading to RV–pulmonary arterial uncoupling [127,128]. Furthermore, left atrial myopathy is a significant factor that contributes to the decline in cardiac function and exacerbates pulmonary vascular disease. This condition is prevalent among patients with HFpEF. It heightens the risk of developing atrial fibrillation or functional mitral regurgitation, both of which are mechanisms through which it adversely affects cardiac hemodynamics [129].

**Figure 1:**
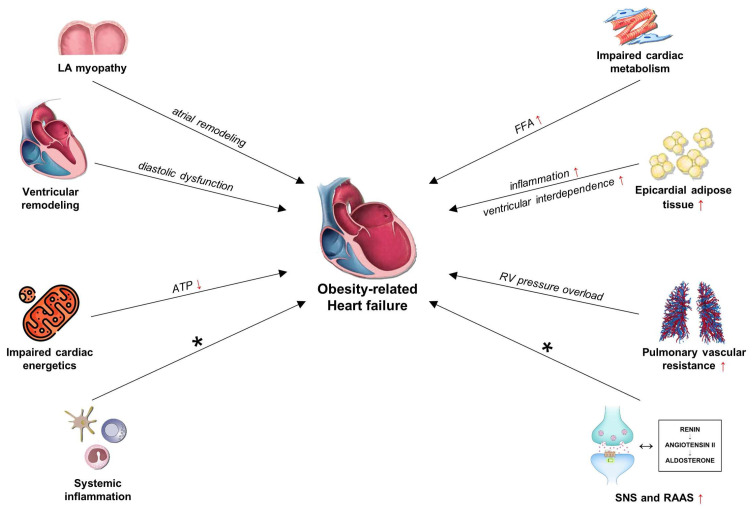
Schematic representation of obesity-induced pathophysiological mechanisms leading to HFpEF, highlighting endothelial dysfunction, systemic inflammation, and metabolic derangements. Abbreviations: FFA: free fatty acids; HF: heart failure; LA: left atria; RAAS: renin–angiotensin–aldosterone system; SNS: sympathetic nervous system. * See text for further information.

Nitrosative stress significantly contributes to HFpEF, particularly in individuals with obesity or metabolic syndrome. Obesity-induced systemic inflammation enhances the production of inducible nitric oxide synthase (iNOS) in cardiac cells, resulting in excessive NO generation. Mitochondrial dysfunction leads to elevated O_2_^−^ levels, the formation of ONOO^−^, and an accumulation of RNS. These RNS induce S-nitrosylation of key proteins, notably IRE1α, impairing the unfolded protein response (UPR) by reducing Xbp1s synthesis [130,131]. The IRE1α–Xbp1s arm of the UPR is particularly significant in mediating the response of cardiomyocytes to many metabolic and proteotoxic stress [132,133]. IRE1α typically cleaves Xbp1 mRNA, resulting in the production of Xbp1s, a transcription factor that regulates lipid metabolism, oxidative stress, and protein quality control [133]. In individuals with overweight, nitrosative stress, particularly the S-nitrosylation of IRE1α, diminishes the efficacy of this pathway, resulting in reduced production of Xbp1s [130]. This decline leads to metabolic dysregulation and facilitates lipid accumulation in heart tissue [130,134]. A significant downstream target of Xbp1s is the transcription factor FoxO1, which affects lipid storage and mitochondrial integrity [133,134]. In optimal conditions, Xbp1s facilitates the degradation of FoxO1 via STUB1, an E3 ubiquitin ligase [134]. Decreased levels of Xbp1s result in increased stability and activity of FoxO1, leading to elevated expression of lipotoxic genes and diminished diastolic function [134,135]. Molecular events elucidate the impact of metabolic stress on the pathogenesis of HFpEF [132]. Researchers are investigating the restoration of the Xbp1s–FoxO1 signaling axis as a potential treatment for HFpEF associated with obesity [134]. Kitakata et al. found that imeglimin mitigates HFpEF-associated molecular dysregulation by inhibiting iNOS induction and restoring Xbp1s expression. This recovers the Xbp1s–STUB1–FoxO1 pathway, resulting in the degradation of FoxO1 and diminished transcription of its lipotoxic targets. Consequently, imeglimin reduces heart adipogenesis and diastolic dysfunction, underscoring its therapeutic promise in obesity-related HFpEF [134]. Obesity has gained particular attention with regard to HFpEF, as a common HFpEF bystander or a direct causative agent of HFpEF (Figure 1) [136].

The mechanisms include the above-noted systemic inflammation, myocardial metabolism and energetics, disturbance in pulmonary vascular tone, atrial myopathy, expansion of epicardial adipose tissue, and also many other indirect mechanisms, with DM being the most important of these [136,137,138,139,140,141,142,143,144]. Except for the indirect effects mediated by atherosclerosis development, it became apparent in the early 1970s that DM also has distinct negative effects on cardiac function, i.e., diabetic cardiomyopathy [145]. With the advent of HFpEF recognition, molecular mechanisms underlying DM-associated HF were extensively explored [143,144]. Diabetic cardiomyopathy is a diverse entity which includes two distinct phenotypes [146]. The dilative phenotype is characterized by complex interaction of hyperglycemia, lipotoxicity, oxidative stress, autoimmune cell destruction, hypoxia, and advanced glycation endproduct deposition that lead to cardiomyocyte cell death and replacement fibrosis, ultimately reducing systolic function and causing clinical HFrEF syndrome. Diabetes contributes to HFrEF by intensifying cardiomyocyte stress and dysfunction through oxidative stress, autoimmune cell destruction, and inhibition of nutrient deprivation signaling and autophagy, as well as AGE deposition, while also activating sodium–hydrogen exchange, which compromises cardiomyocyte survival [144,147]. Conversely, restrictive phenotype is characterized by reactive interstitial fibrosis, endothelial dysfunction, and cardiomyocyte hypertrophy, all of which contribute to diastolic dysfunction and thus, HFpEF development. The mechanism underlying HFpEF also involves an increase in the expansion of visceral, particularly epicardial, adipose tissue, which may contribute to heart inflammation, microcirculatory dysfunction, and fibrosis [147]. Evidence-based medical therapy varies between HFrEF and HFpEF, with diuretic therapy being the primary treatment option for HFpEF until six years ago. Recent years have seen a significant shift, with numerous studies demonstrating the clinical benefits of SGLT2 inhibitors and GLP-1 receptor agonist therapy in patients with HFpEF [148,149,150,151,152,153,154,155]. Empagliflozin and dapagliflozin were associated with reductions in cardiovascular mortality among patients with HFpEF, independent of diabetes mellitus, and both agents exhibited good tolerability [149,150,151,152]. Furthermore, 12-week treatment with dapagliflozin resulted in significant improvements in patient-reported symptoms, physical limitations, and exercise function [151]. SGLT2 inhibitors exhibit numerous benefits, including decreased blood pressure, reduced plasma volume, lower vascular stiffness, and resistance, as well as reductions in visceral and subcutaneous adipose tissue and body weight [156,157,158]. Additionally, the GLP-1 receptor agonist semaglutide has been shown to alleviate symptoms of heart failure and enhance exercise capacity in patients with obesity-related heart failure and HFpEF, regardless of their diabetes status. It also demonstrated favorable outcomes concerning the incidence of major adverse cardiovascular events, including cardiovascular death, nonfatal myocardial infarction, or nonfatal stroke, as well as heart failure composite outcomes, defined as cardiovascular death, hospitalization, or emergency room visits for heart failure [154,155,159]. Targeting cardiac inflammation has been proposed as a potential treatment strategy for heart failure. SGLT2 inhibitors and GLP-1 receptor agonists exhibit anti-inflammatory effects [160,161,162]. Nonetheless, several other drugs are currently under investigation and have not yet been incorporated into official treatment guidelines for these patients [163]. Targeted cytokine therapies, including anakinra (an IL-1 receptor antagonist), colchicine, statins, omega-3 fatty acids, and vagus nerve stimulation, currently lack robust evidence for the treatment of HFpEF. However, clinical trials are ongoing for several of these therapies, such as anakinra, colchicine, and AZD4831 (a myeloperoxidase inhibitor), to evaluate their therapeutic efficacy against HFpEF and HFmrEF [163,164,165].

Kidneys are not spared the detrimental effects of nutritional excess and obesity [166]. Apart from the well-established association between DM, hypertension, and chronic kidney disease (CKD) etiopathogenesis, evidence suggests that obesity may contribute to CKD development and progression beyond indirect effects mediated by DM and hypertension [167]. The mechanisms explaining obesity-related kidney disease include glomerular hyperfiltration, RAAS activation, lipotoxicity, insulin resistance, and changes in intestinal flora [167]. The previously mentioned therapy involving SGLT-2 inhibitors and GLP-1 receptor agonists has demonstrated significant benefits in patients with CKD, particularly in the context of treating metabolic syndrome [168,169].

Our current work has some limitations. Despite the comprehensive literature review, there remains a possibility that certain pertinent works in this domain were not reported due to factors such as database search limitations and publication language. We sought to mitigate this risk by utilizing multiple databases, employing comprehensive search terms, reviewing the reference lists of significant papers, and not limiting inclusion based on publication date or study design. Moreover, we endeavored to incorporate and synthesize a substantial array of research employing diverse methodological approaches, examining various populations, and investigating different outcomes. This complicates the derivation of conclusions; however, we distinctly emphasize and address significant methodological disparities in the interpretation of the results, thereby mitigating the risk of erroneous generalizations. Lastly, it is essential to note that research with negative or inconsistent outcomes is often not published, thereby introducing bias in the interpretation of predominantly positive findings.

**Figure 2:**
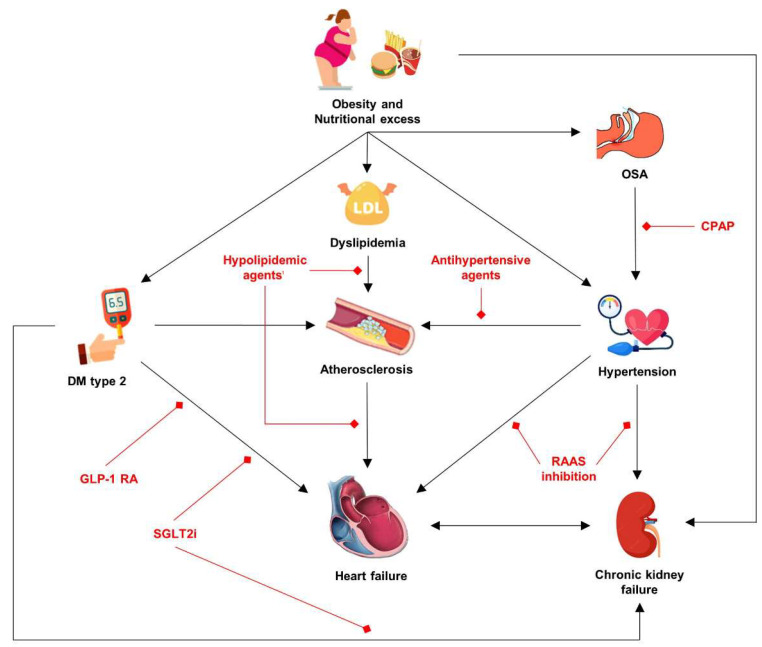
Integrated model of the cardio-renal-metabolic syndrome in obesity, with therapeutic targets highlighted in red. Abbreviations: CPAP: continuous positive airway pressure; DM: diabetes mellitus; GLP-1 RA: glucagon-like peptide-1 receptor agonists; RAAS: renin–angiotensin–aldosterone system; SGLT2i: Sodium/glucose cotransporter-2 inhibitors.

Overall, it can be concluded that oxidative stress and excessive caloric intake affect the development and progression of cardio-renal-metabolic syndrome, through the several pathophysiological mechanisms described. In Figure 2, we highlight the main components of cardio-renal-metabolic syndrome, as well as individual therapeutic options for each component. In other words, obesity causes systemic disease which can vary in clinical phenotype depending on which organ system is predominantly affected.

## 5. Conclusions

Abundant epidemiological data places overfeeding and obesity as a pivotal risk factor in DM, hypertension, HF, and many other diseases in the cardiometabolic spectrum as well. In fact, overconsumption of calories seems to be detrimental even during the prenatal stage. Animal and human studies consistently demonstrate that oxidative stress is a critical effector arm in this setting. The detrimental effects of ROS and RNS are multifactorial and include DNA damage, activation of transcription factors, increase in inflammatory factors, dysfunction of mitochondria, damage to proteins and lipids, and epigenetic alterations. Unfortunately, therapeutic interventions aimed at reducing oxidative stress have mostly yielded unfavorable results, probably because of our inability to reduce oxidative stress by the right amount at the right time. A separate issue in the obesity pandemic is the fact that the problem is commonly not adequately perceived by patients owing to mixture of psychosocial and physiological factors. Hence, except for the quest for therapeutic interventions for cardiometabolic diseases, we should seek the ways by which we can prevent or at least diminish the obesity pandemic.

## Data Availability

Not applicable.
